# Understanding the Magnetic Microstructure through Experiments and Machine Learning Algorithms

**DOI:** 10.1021/acsami.2c12848

**Published:** 2022-10-21

**Authors:** Abhishek Talapatra, Udaykumar Gajera, Syam Prasad P, Jeyaramane Arout Chelvane, Jyoti Ranjan Mohanty

**Affiliations:** † Nanomagnetism and Microscopy Laboratory, Department of Physics, Indian Institute of Technology Hyderabad, Kandi, Sangareddy502285, Telangana, India; ‡ Consiglio Nazionale Delle Ricerche, CNR-SPIN c/o Università“G. D’Annunzio”, Chieti66100, Italy; § Defence Metallurgical Research Laboratory, Kanchanbagh, Hyderabad500058, India; ∥ Chemistry Department, University of Turin, Via Pietro Giuria, 7, Torino10125, Italy

**Keywords:** magnetic domains, magnetic force microscopy, convolutional neural network, micromagnetic simulation, machine learning

## Abstract

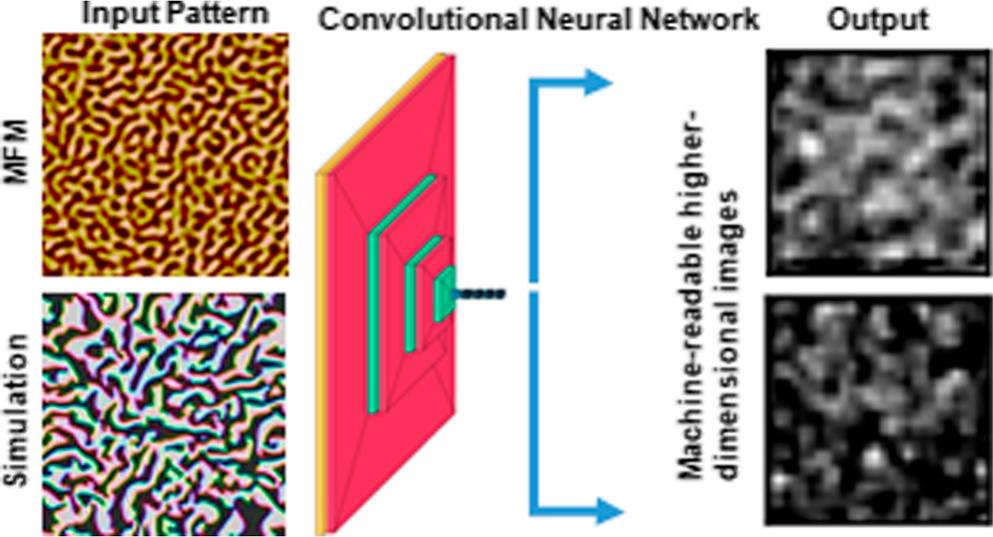

Advanced machine learning techniques have unfurled their applications in various interdisciplinary areas of research and development. This paper highlights the use of image regression algorithms based on advanced neural networks to understand the magnetic properties directly from the magnetic microstructure. In this study, Co/Pd multilayers have been chosen as a reference material system that displays maze-like magnetic domains in pristine conditions. Irradiation of Ar^+^ ions with two different energies (50 and 100 keV) at various fluences was used as an external perturbation to investigate the modification of magnetic and structural properties from a state of perpendicular magnetic anisotropy to the vicinity of the spin reorientation transition. Magnetic force microscopy revealed domain fragmentation with a smaller periodicity and weaker magnetic contrast up to the fluence of 10^14^ ions/cm^2^. Further increases in the ion fluence result in the formation of feather-like domains with a variation in local magnetization distribution. The experimental results were complemented with micromagnetic simulations, where the variations of effective magnetic anisotropy and exchange constant result in qualitatively similar changes in magnetic domains, as observed experimentally. Importantly, a set of 960 simulated domain images was generated to train, validate, and test the convolutional neural network (CNN) that predicts the magnetic properties directly from the domain images with a high level of accuracy (maximum 93.9%). Our work has immense importance in promoting the applications of image regression methods through the CNN in understanding integral magnetic properties obtained from the microscopic features subject to change under external perturbations.

## Introduction

Magnetic thin films exhibiting perpendicular magnetic anisotropy (PMA) are of technological importance and promising candidates for spintronic nanodevices in the context of ultrahigh density magnetic storage,^1^ fast memory applications,^2^ and nanosensors.^3^ At favorable atomic ordering, ultrathin stacking of Co with Pd or Pt displays PMA. For magnetic thin films and multilayers with PMA, the information about magnetic microstructure is extremely important to understand the magnetization reversal, which is governed by energetics. The net magnetic energy (*E*_total_) for a thin film can be represented as *E*_total_ = *E*_K_ + *E*_A_ + *E*_D_ + *E*_H_, where *E*_K_, *E*_A_, *E*_D_, and *E*_H_ represent the anisotropy energy, exchange energy, magnetostatic energy, and Zeeman energy, respectively. The minimization of *E*_K_ tries to drive the magnetization along the easy axis, which can be tuned with thickness, growth parameters, and route of processing of the materials for a real system. *E*_A_ gets minimized when the spins align in parallel and thus prefer the formation of a single domain. On the contrary, *E*_D_, primarily determined by the saturation magnetization and the shape of the magnetic structure (important in the case of patterned structures), opposes the single domain formation. Thus, in the absence of an external magnetic field (*E*_H_ = 0), the minimization of the total energy terms leads to the formation of magnetic domains.^4^ In reality, it is complicated to predict the simultaneously changing competing magnetic interactions that result in various domain morphologies for perpendicularly magnetized systems.^5−7^ Now, the characteristic features of magnetic domains in multilayer (ML) films can be controlled by different factors such as growth conditions,^8^ the thicknesses of the constituent layers, as well as the application of external stimuli, viz., magnetic field, heat treatment,^9^ laser,^10^ and ion irradiation.^11^ Moreover, the tunable exchange coupling in the exchange spring magnets can significantly control the magnetic microstructure.^12,13^ Thus, modeling of process-induced magnetic modifications has also been proven to be important in designing highly controlled experiments and predicting new materials and methods.

In recent times, machine learning techniques have been proven to be an important tool to understand material systems with interdependent and simultaneously variable material properties. In this paper, we are going to address the application of the convolutional neural network (CNN) to understand the modifications of magnetic domains in a perpendicularly magnetized multilayer, which has been observed experimentally by using ion-beam irradiation. Of late, advanced machine learning techniques have acquired immense importance in interdisciplinary research, such as in microstructure optimization,^14^ prediction of a magnetic field,^15^ phase transition,^16^ magnetic grain size study,^17^ modeling magnetic domains,^18,19^ relation between different magnetic chiral states,^20^ prediction of effective magnetic spin configurations,^21,22^ 2D metal–organic frameworks with high magnetic anisotropy,^23^ and different components of Hamiltonian including the Dzyaloshinskii–Moriya interaction (DMI),^24^ using different deep learning and machine learning methods. From the point of view of atomistic magnetism, researchers^21,22^ have tried to estimate and analyze various components of Hamiltonian, such as exchange constant, anisotropy constant, and DMI, using different CNNs.^25^ The advanced CNN methods showed effectiveness and accuracy in different research domains. However, these methods generally require a large data set^26^ to properly train and test the model, which might stand in the way of directly using these methods in analyzing regular experimental data.

From the experimental point of view, ion-beam irradiation is a popular and viable technique to tune magnetic properties, associated with a locally induced structural imperfection or intermixing, relevant in the context of magnetic MLs.^27^ The ion energy and fluence (number density) can be separately adjusted to control the depth and lateral extent of the irradiation-induced effects, which leads to the modification of magnetic properties. The ions lose energy during their passage through the material, which is either spent in displacing the target atoms by elastic collision (nuclear stopping) or exciting the atoms by inelastic collisions (electronic stopping).^28^ A large variety of studies exists on ion-beam induced modifications in magnetic ML, either in the context of patterning^29^ or depth-resolved structural modifications.^30,31^ The creation of graded anisotropy media by domain wall positioning has also been reported using ion irradiation.^32^ Ion-induced modification of magnetic properties with depth-resolved structural studies in Co/Pt ML has been reported.^33,34^ An interesting study on the investigation of magnetic domains after ion beam irradiation was performed by Trassinelli et al., where local microscopic features of domains were highlighted in the vicinity of the ferromagnetic to paramagnetic phase transition temperature for Mn–As thin films.^35^ Co/Pt ML^36^ and soft magnetic FeCoSiB thin film^37^ systems have also been investigated in order to study the behavior of magnetic domains in response to light ions. Swift heavy ion-induced modification of magnetization dynamics,^38^ lattice distortion^39,40^ and spin reorientation^41^ are also topics of contemporary research interest. Of late, deterministic generation^42^ and precise tunability of skyrmion density^43^ have been achieved using energetic ions. From the point of view of ferrimagnetic thin films, the compensation point and magnetic anisotropy were successfully engineered in Gd–Fe and Tb–Fe thin films using He^+^, and Ne^+^ ions with various fluences.^44^ Interestingly, magnetic domains were engineered without domain walls in Tb/Co ML with the bombardment of He^+^ ions.^45^ Our recent works on Ar^+^-induced modifications of magnetic properties in the amorphous Tb–Fe–Co thin films^46^ and Tb–Fe/Gd–Fe/Tb–Fe trilayer^47^ highlighted the tunability of magnetic domains correlated with the structural changes in the vicinity of the spin reorientation transition.

The focus of this paper is to understand the modifications of magnetic properties with a special interest in nanoscale magnetic domains, displaying a transition from the maze-like pattern in pristine condition to feather-like domains as a function of the fluence (*F*) and energy (*E*) of Ar^+^ ions. Micromagnetic simulations were performed to record the variations of magnetic domains with micromagnetic input parameters. Furthermore, we have proposed a model using a CNN-based image regression technique to identify and recognize various features of magnetic domains correlated with the magnetic properties (micromagnetic input parameters). A large set of simulated domain images was used to train and test the neural network (NN) which determines the magnetic properties of the unknown (out of the sets used for training) domain images with higher accuracy. Our work directs a novel route for the quantitative analysis of microscopic domain images using artificial intelligence.

## Methods

### Experiments

Co/Pd ML films with the configuration of Si (substrate)/Ta (30)/Pd (30)/[Co (*t*_Co_)/Pd (8)]_×50_/Pd (12) (the numbers in parentheses indicate thicknesses in Å) have been deposited by ultrahigh vacuum DC magnetron sputtering at a working pressure of 1.35 × 10^–3^ Torr of Ar. The ion-beam irradiation was performed on the films with maximum PMA, obtained with a specific *t*_Co_. The ion-irradiation process was carried out under high vacuum (2 × 10^–6^ Torr), normal to the film surface using Ar^+^ ions of energies 50 and 100 keV with varying fluences, ranging from 0.5 × 10^14^ to 3.3 × 10^16^ ions/cm^2^. The time of irradiation (*t*) controls the ion-fluence following the relation , where σ represents the area of irradiation (1 cm^2^), g denotes the charge state of the ion (+1), *I* denotes the beam current (2 μA), and *e* is the electronic charge. Table 1 describes the nomenclature of the films with different values of fluence. Simultaneous atomic and magnetic force microscopy (AFM and MFM) were carried out to observe the topography and magnetic domains. Magnetization reversal has been studied with a vibrating sample magnetometer (VSM) by applying the external magnetic field along the in-plane (IP) and out-of-plane (OOP) directions with respect to the film surface. Cross-sectional transmission electron microscopy (XTEM) was carried out for selective samples employing high voltage (1250 kV) electron microscopy. Spatial mapping of the corresponding elements was performed using energy-dispersive spectroscopy (EDS) of the X-ray associated with the FEI Titan scanning transmission electron microscope. The depth-resolved structural investigation was complemented by X-ray reflectivity (XRR) measurements.

**Table 1:** Nomenclature of the Samples and Comparison of *M*_R_, *H*_c_, *K*_eff_, and *R*_q_ with Variable Ion Fluences

*F* in ions/cm^2^	name	*M*_R_ in emu/cm^3^ OOP (IP)	*H*_c_ in Oe OOP (IP)	*K*_eff_ in erg/cm^3^ (×10^6^)	*R*_q_ in nm
0	pristine	1200 (120)	880 (440)	3.5	0.4
0.5 × 10^14^	A1	260 (395)	243 (244)	–0.9	0.5
1 × 10^14^	A2	47 (424)	188 (268)	–2.7	0.5
3.3 × 10^14^	A3	56 (921)	201 (80)	–5.3	0.4
1.0 × 10^15^	A4	68 (68)	230 (226)	–0.2	1.0
3.3 × 10^15^	A5	86 (783)	245 (75)	–5.0	1.4
1.0 × 10^16^	A6	98 (779)	234 (85)	–4.9	1.0
3.3 × 10^16^	A7	58 (483)	162 (88)	–3.3	2.2

### Micromagnetic Simulation

Micromagnetic simulations of magnetic domains were performed using MuMax3 software.^48^ The Co/Pd ML is considered as a single magnetic layer with an effective anisotropy constant (*K*_eff_) and effective thickness (*t*_eff_) of the magnetic layers, comparable to the multilayer. The input parameters are close to the values reported in the literature,^49^ that is, exchange constant (*A*_ex_) = 2.3 μerg/cm, saturation magnetization (*M*_s_) = 1 kemu/cm^3^, and *K*_eff_ = 10 Merg/cm^3^. The simulation temperature (*T*) and damping constant (α) were 300 K and 0.9, respectively. Cubic meshes of volume (4 nm)^3^ are used for the discretization of the total area of simulations (∼2 μm × 2 μm) with *t*_eff_ = 16 nm. The simulations started from arbitrary initial spin configurations and run for 100 ns to obtain energy-minimized stable configuration of magnetization following Landau–Lifshitz–Gilbert equation.^50,51^ Here our main focus is to analyze the domain images which essentially signify the spatial variation of the overall magnetization (*M*).

### Building and Training of CNN

In this article, the CNN-based image regression technique is used. We have used different popular deep learning (DL) models as mentioned below.

- Cutsom multilayer perceptron model using Tensorflow.^52^
- Residual NN architectures ResNets with and without pretrained models.^53^
- VGG16 with improved (3 × 3) convolution filters^54^
- DenseNet, which utilize dense connections between layers through dense blocks.^55^
- One of the classic DL algorithms: AlexNet^56^
- EfficientNet uniformly scales all dimensions of depth, width, and resolution using compound coefficients.^57,58^

The custom architecture of CNN contains a multilayer perceptron with a batch normalization layer followed by a dense NN. For optimization, during the training of CNN, the default function Adam is used, which is the first-order gradient-based optimization of stochastic objective functions. For the pretrained ResNets models, we have used Fastai and PyTorch libraries to train and predict the different magnetic properties from the simulated domain images. We have also compared the performance using different models such as ResNet-18 and ResNet-34 comprising 18 and 34 layers, respectively. Overfitting is prevented by employing the default early-stopping algorithm. The learning rate is set to 0.001 with a strategy to reduce the learning rate when the error stops decreasing with several steps. Furthermore, early stopping is implemented in a way that it decides to stop training based on accuracy improvement. The fit one cycle method was used for the dynamic learning rate implemented in PyTorch. We have also checked our model using pretrained weights.

In order to create a large data set for training the NN, we have used 960 simulated images, obtained by realistic variations of four micromagnetic input parameters, viz., *A*_ex_ (range: 0.1–2.7 μerg/cm), α (range: 0.825–0.925), *K*_eff_ (range: within the order of 10^4^ to 10^9^ erg/cm^3^), and *T* (range: 300–1000 K). The considered range of parameters makes the data sets versatile by incorporating various types of magnetic domains of different characteristic length scales and spatial features. Physically, the variation of *A*_ex_ and *K*_eff_ results in a change in domain wall energy (proportional to ). The variation in temperature introduces thermal agitation that results in fluctuation of magnetization near the boundary between two oppositely magnetized domains. α controls the relaxation of magnetization. The entire data set was divided into three different groups, as mentioned below.

- The first group was used for training the model, using 63% (605) of the total images.
- The second group was utilized for the validating and rearrangement of weights, using 7% (67) of the total images.
- The third group was used to test the model, using 30% (288) of the total images.

It is worth mentioning that the images used for validating were also used during the training process. However, the images used for testing the model were never exposed to the machine during training.

## Results and Discussions

### Pristine Co/Pd Multilayers

**Figure 1:**
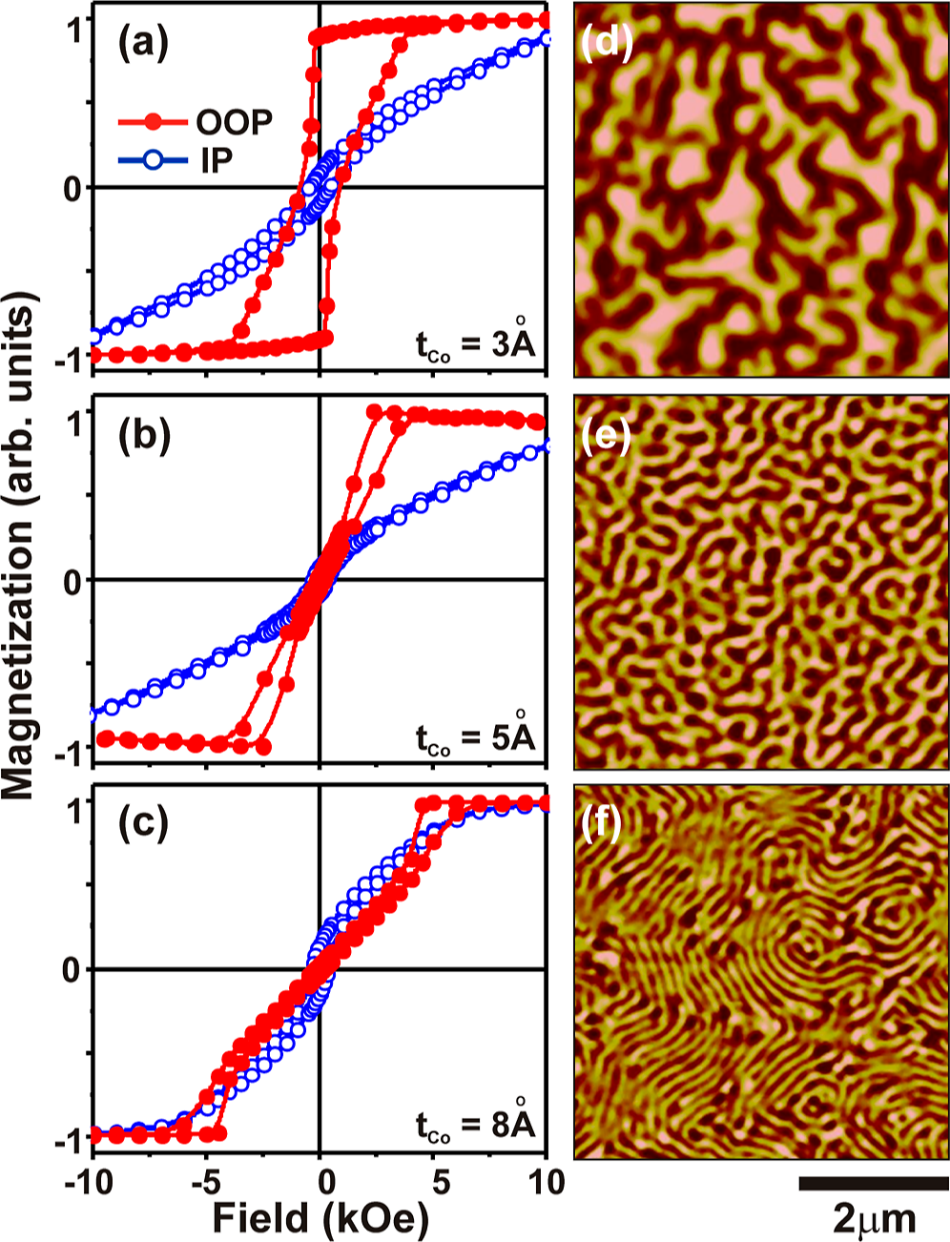
The in-plane and out-of-plane hysteresis loops for Co/Pd ML at different *t*_Co_ are shown in (a–c) along with the corresponding MFM images of magnetic domains in the as-deposited state, shown in (d–f) for *t*_Co_ = 3, 5, and 8 Å, respectively.

Co/Pd ML films were deposited with three different *t*_Co_ to observe the variation in PMA. Figure 1a–c represents the OOP and IP hysteresis loops for the values of *t*_Co_ of 3, 5, and 8 Å, respectively, with a fixed Pd layer thickness of 8 Å and number of Co/Pd repeats of 50. Additionally, Ta and Pd were used as the buffer layers to aid the PMA. For *t*_Co_ = 3 Å (Figure 1a), an almost square hysteresis loop can be observed with high remanence (*M*_R_) and coercivity (*H*_c_) along the OOP direction, which is the indication of strong PMA. On the other hand, slanted hysteresis loops are observed with the increase in *t*_Co_, as shown in Figure 1b,c, which indicate the onset of nucleation of reversed domains at comparatively higher magnetic fields. A quantitative comparison shows that the OOP *H*_c_ is 884 (±10) Oe for *t*_Co_ = 3 Å and decreases to 161 (±10) Oe and 205 (±10) Oe for *t*_Co_ = 5 and 8 Å, respectively, whereas the OOP saturation field (*H*_s_) varies to 5, 5.5, and 7 kOe, as shown in Figure 1a–c, respectively. The value of the nucleation field (*H*_N_) is closely related to the ratio of saturation magnetization (*M*_s_) to *M*_R_, as both the quantities play a decisive role in magnetization reversal. A high *M*_R_/*M*_s_ of 90% and *H*_N_ of around −200 Oe in Figure 1a clearly indicate that the magnetization reversal is dominated by the rapid domain wall motion.^59^ The values of *H*_N_ are close to 2.4, and 4.5 kOe along with weak *M*_R_/*M*_s_ of 4 and 2.9% for Figure 1b,c, respectively, which confirm that nucleation, propagation, and annihilation of the reverse domains lead to the magnetization reversal of these films. The IP loops for all three films cannot be brought up to saturation within the limited field of the VSM measurements. Thus, the magnetization studies confirmed that all the ML displays a predominant easy axis along the OOP direction among which the strongest PMA can be observed with *t*_Co_ = 3 Å. Microscopic investigation of *t*_Co_-induced variation of PMA was performed using MFM. All the pristine Co/Pd ML displays a uniform featureless surface (not shown) with a root mean square roughness (*R*_q_) of less than 1 nm, and the domain imaging has been performed in the as-deposited condition of the films before exposure to an external magnetic field. Periodic, nanoscale magnetic domains can be observed in Figure 1d–f for *t*_Co_ = 3, 5, and 8 Å, respectively. Maze-like domains with strong OOP contrast with an average lateral size of 434 (±12) nm can be observed in Figure 1d. The domain size reduces to 176 (±2) nm, and 88 (±2) nm for Figure 1e,f, respectively. The reduction in the OOP contrast is also clear from the normalized MFM images with a transition to thinner stripes domains for the highest *t*_Co_. The effective magnetic anisotropy (*K*_eff_) for magnetic multilayers can be expressed as , where *K*_v_ and *K*_int_ represent the volume and interface anisotropy constants, respectively, with *t* as the effective thickness of the magnetic layers. The magnetic properties of the ML can be modified with the variation of the number of stacking or by varying the thickness of the constituent materials.^60,61^ The increase in the effective thickness reduces the contribution of interfacial anisotropy energy and simultaneously increases the volume anisotropy energy. In this competition, whenever both the interface and volume anisotropy energies become comparable, the system breaks into multiple domains. From the results of Figure 1, it can be confirmed that the domain nucleation is more favorable at higher *t*_Co_, which essentially reduces the PMA of the systems, and hence, the highest contribution of interface anisotropy energy at the lowest *t*_Co_ is considered to play a major role behind the strong PMA.

### Effect of Ion Irradiation

#### Magnetic Characterizations

**Figure 2:**
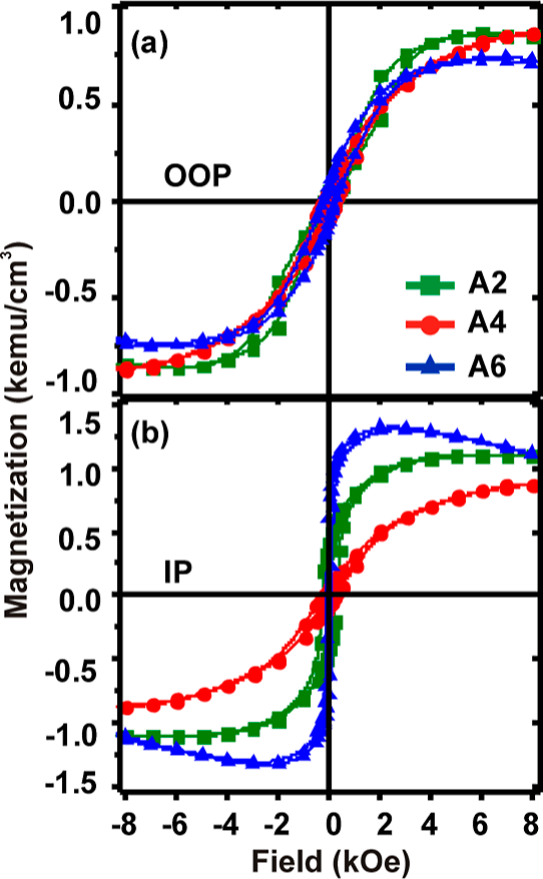
Hysteresis loops of the irradiated films with the applied field along the (a) out-of-plane and (b) in-plane directions.

Owing to the possession of strong PMA, the films with *t*_Co_ = 3 Å have been used for the ion irradiation studies. The integral magnetic response of the irradiated films has been characterized with the OOP and IP hysteresis loops, as depicted in Figure 2a,b, respectively, for the selected samples. It can be clearly understood from Figure 2 that the maximum magnitude of magnetization detected in the IP mode is larger than that in the OOP mode, in contrast to the case of the pristine film, shown in Figure 1a. This essentially indicates that irradiation with 50 keV ion energy triggers a spin reorientation transition (SRT) from OOP to IP with respect to the film surface. The OOP loops become slanted with reduced *M*_R_/*M*_s_ and a higher saturation field, whereas the IP loops gain *M*_R_ with a lower saturation field. At higher fluence of irradiation, the collision between the larger number of energetic ions and the film surface may result in the formation of surface defects that act as the pinning sites for the domain wall and restrict the rapid domain wall motion, as interpreted from the hysteresis loops (Figure 1a) of the pristine film. The values of IP and OOP *M*_R_, *H*_c_, and *K*_eff_ have been recorded in Table 1 for all the irradiated films. Although the variations of the parameters are not very systematic, a huge increase in IP *M*_R_ is to be noticed with respect to its OOP counterpart. The IP loops gain squareness with a decreased IP coercivity, which shows variation close to the error limit of ± 5 Oe at higher *F* values. This behavior is also reflected in the *K*_eff_ values. Most importantly, the negative values for *K*_eff_ essentially indicate the presence of easy-plane anisotropy, originating due to the SRT induced by ion irradiation. The hysteresis loops for the irradiated samples at various fluences with *E* = 100 keV are presented in Figure S1 of the Supporting Information.

**Figure 3:**
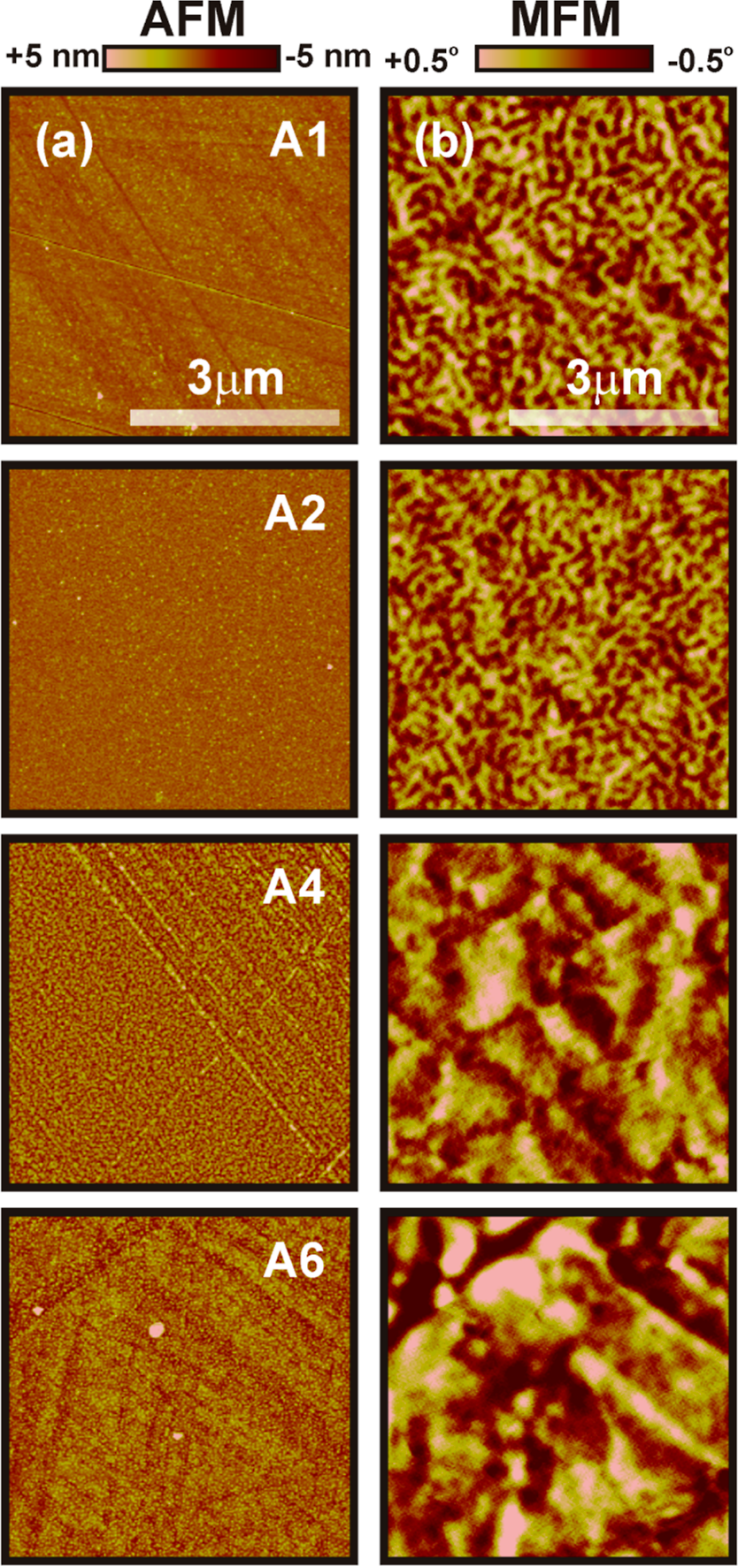
Normalized (a) AFM images with simultaneously captured (b) MFM images for the films irradiated with 50 keV of Ar^+^ ions at various fluences (specified by the nomenclature). The scale bar is the same for all the images.

The simultaneously recorded normalized AFM and MFM images are depicted in Figure 3a,b, respectively, for the irradiated films. The *R*_q_ values, listed in Table 1, are determined from the AFM images and show an overall increasing trend with the increase in *F*. This can be connected with the ion-induced surface damage with irradiation for longer times at higher *F*. In addition to that, the growth of irregularly distributed granular topographic features has been observed with high-resolution AFM imaging (not shown here), where the maximum grain size appears to be 141 nm with a distribution error of ±50% for the A6 film. The MFM images (Figure 3b) for A1 and A2 display a maze-like domain pattern with alternate dark-bright contrast, reduced with respect to that of the pristine film, as shown in Figure 1d. The average domain size is estimated to be 150 (±4) nm and 126 (±3) nm for A1 and A2, respectively. A3 is seen to be the threshold where a domain pattern with this in-plane correlation length no longer exists. Instead, feather-like domains are observed for A3 and A4. Finally, the magnetic contrast drops significantly for A6 and topographic interference starts to appear in the MFM images due to the increased height of the topographic features. Two distinct factors are relevant for this domain transformation. The first one is ion energy which determines the penetration depth of the ions inside the film. This gives rise to intermixing at the interfaces by modifying the periodicity of the ultrathin stacking. Hence, the effective anisotropy is expected to decrease,^33^ causing a reduction in the domain size and the associated magnetic contrast, as observed in the case of A1 and A2. The other effect is fluence, which is responsible for the extent of modification of surface properties such as defects and pinning sites for domain walls. The observed feather-like domains can be explained based on the IP magnetization distribution and transport of the magnetic charge toward the surface, which is achieved by a small deviation from the IP magnetization, similar to the formation of cross-tie walls.^62^ The modified domain morphologies can be different for different material systems and choice of ions.^63^ The surface roughness of the films increases with higher ion energy, and the fragmented maze-like domains could not be observed for the irradiated samples with *E* = 100 keV; the corresponding AFM and MFM images are presented in Figure S2 of the Supporting Information. Thus, variation in the domain size and pattern can be tuned *via* locally competing anisotropies, which create a strong force for the SRT toward the IP direction.

#### Depth-Resolved Structural Characterizations

**Figure 4:**
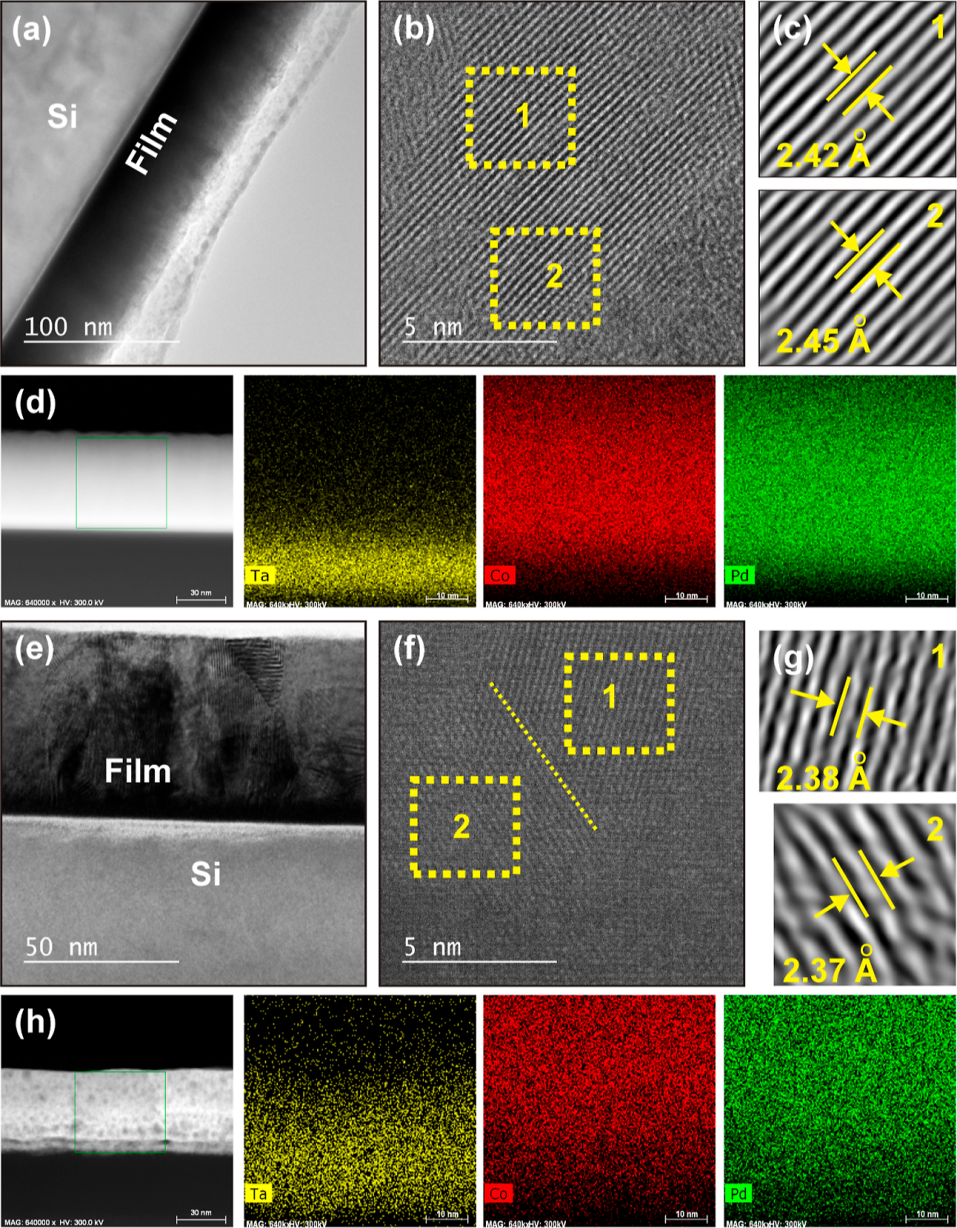
(a,e) cross-sectional TEM images, (b,f) HRTEM images, and (c,g) IFFT images for the pristine Co/Pd ML and A7 films, respectively. (d,h) EDS mapping of the distribution of the corresponding elements in the pristine and A7 films, respectively.

The depth-resolved structural studies were performed with XTEM. First, we have shown the TEM image for the pristine [Co (3 Å)/Pd (8 Å)]_×50_ multilayer (with highest PMA) in Figure 4a, which shows a multilayer film on top of the single-crystalline Si substrate. A high-resolution (HR) TEM image is depicted in Figure 4b, clearly displaying the presence of lattice fringes. Inverse Fourier filtered transform (IFFT) was performed selectively at different regions (regions 1 and 2 are indicated by yellow boxes for clarity) of Figure 4b. The average value of the d-spacing was estimated from the line scans over the IFFT images shown in Figure 4c and turned out to be around 2.43 (±0.02) Å. This lattice spacing can be attributed to the *fcc*(111) crystal orientation of the Co/Pd system that appears due to the lattice averaging of Co and Pd, and therefore, can be tuned with *t*_Co_.^64,65^ It is always intriguing to analyze the depth-resolved elemental mapping for the case of ultrathin multilayer stacks, which provides information about the interfaces. The EDS technique was used to perform the elemental mapping of the constituent elements along the specified region (box), shown in the high-angle annular dark-field scanning transmission electron microscopy (HAADF-STEM) image in the extreme left of Figure 4d. Thereafter, the mappings for Ta, Co, and Pd are shown from left to right which well justifies Ta as the buffer layer. However, Co and Pd are observed everywhere in the selected region of mapping, which suggests the possibility of alloying at the interfaces of the corresponding ultrathin layers in the pristine state. Yang et al. reported the absence of the multilayer feature when thicknesses of both Co, and Pd layer reach around 5 Å.^66^ The XTEM and HRTEM images of the sample, irradiated at the maximum fluence (A7), are shown in Figure 4e,f, respectively. Unlike the pristine sample, Figure 4e displays the presence of irregular and aperiodic stripes that could probably be the footprint of the bombarded ions. Two regions with different textures have been indicated in Figure 4f, where the position of the dotted line could be the grain boundary in the polycrystalline film. The average *d*-spacing estimated from the IFFT images of Figure 4g is around 2.37 (±0.06) Å, indicating no change in the *fcc*(111) growth direction. The estimations of *d*-spacing are comparable to those values, mentioned by Barton et al.^67^ The HAADF-STEM image of Figure 4h shows the presence of bubble-like features, which might be the Ar bubbles, appearing after irradiation. However, we could not confirm the presence of Ar with EDS due to its lighter atomic weight. The alloying of Co and Pd is clear from the EDS mapping, shown in Figure 4h, which also suggests the possibility of the diffusion of Ta in the multilayer.

**Figure 5:**
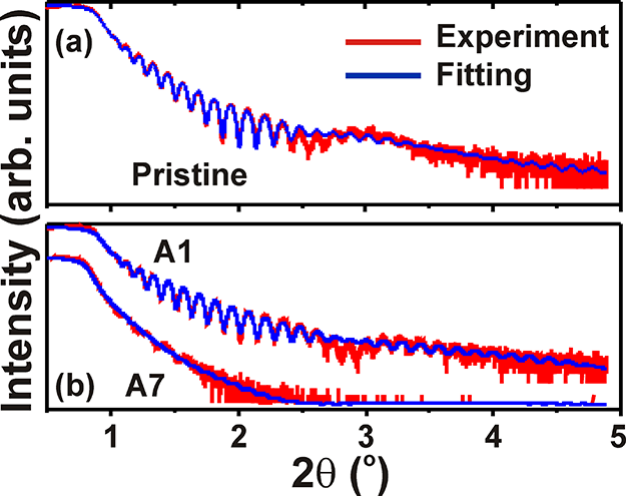
Experimental and fitted XRR spectra for (a) pristine and (b) irradiated A1 and A7 multilayer.

The XTEM studies on the depth-resolved microstructure have been complemented with XRR. Comparative XRR spectra for the pristine Co/Pd ML and the irradiated films with minimum (A1) and maximum (A7) fluence are presented in Figure 5. All the spectra have been fitted with multilayer models, relevant to the experimentally prepared samples. The best-fitted data along with the experimental spectra for the pristine film are shown in Figure 5a, which confirms the presence of interfacial diffusion of Co and Pd, as explained from the elemental mappings in Figure 4d. The fitted model suggests the formation of the [Co (1.8 Å)/CoPd (1.13 Å)/Pd (7.1 Å)]_×50_ ML in the pristine state (Figure 5a), and [Co (1.3 Å)/CoPd (3.16 Å)/Pd (5.96 Å)]_×50_, and [CoPd (10.82 Å)/Pd (3.34 Å)]_×50_ for A1 and A7, respectively, in Figure 5b. The highest ion fluence in A7 results in complete diffusion of Co along with higher interfacial and surface roughnesses, and consequently, the XRR intensity falls rapidly at lower 2θ values. The XRR results follow similar trends when the ion energy increases to 100 keV at the same fluences (Figure S3 of the Supporting Information). Thus, the XRR studies explain the broadening of interface width and roughness with increasing energy and fluence of Ar^+^ ions.^68^ The interface quality and magnetic properties change significantly due to reduced symmetry breaking at the Co/Pd interfaces.^67^ It is worth mentioning that consideration of a thin native oxide layer of SiO_2_ above the Si substrate makes the fitting model more accurate and viable with the real samples.

### Simulated Domain Configurations

**Figure 6:**
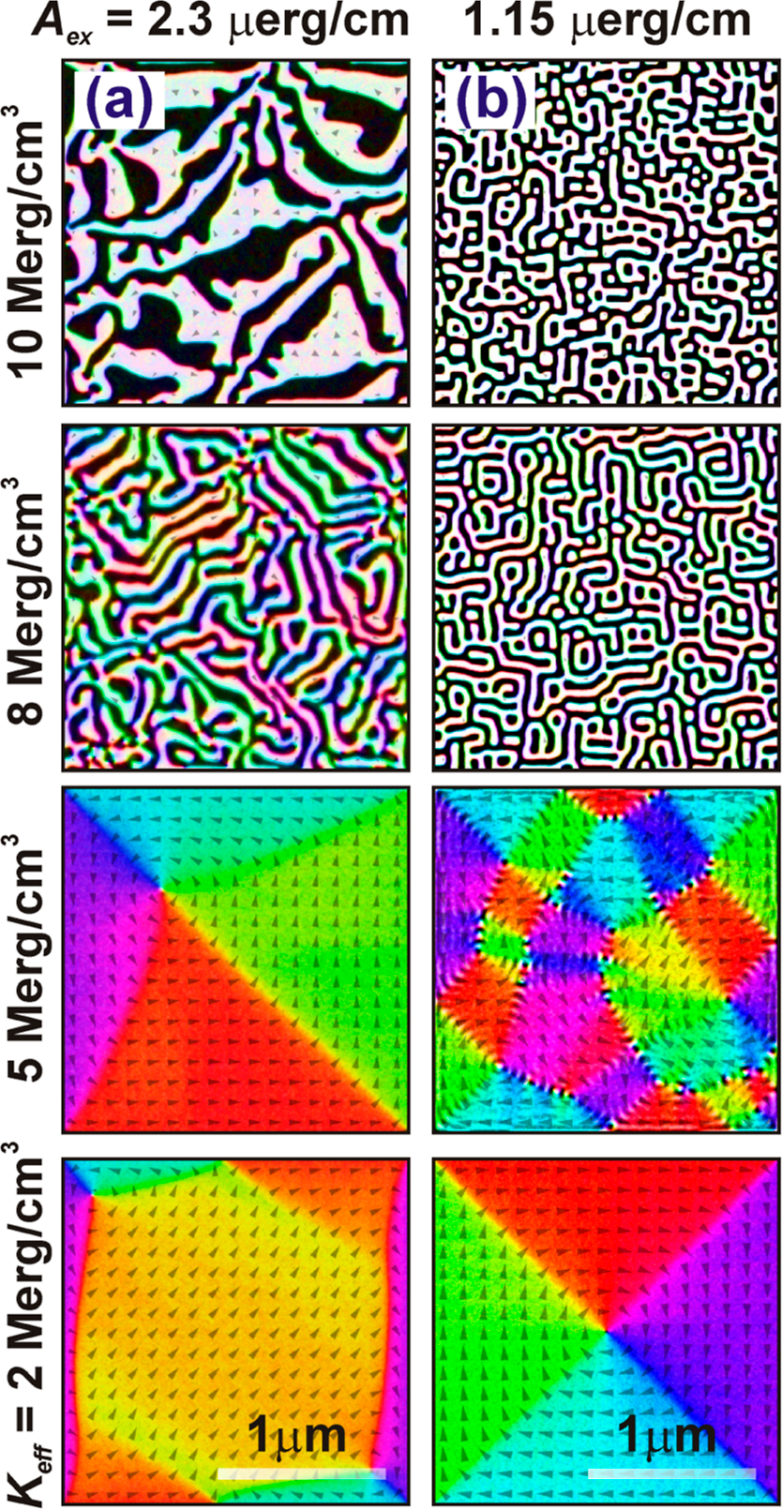
Simulated domain images with different *K*_eff_ (mentioned in the left column) with *A*_ex_ of (a) 2.3 and (b) 1.15 μerg/cm. The scale bar is the same for all the images.

Detailed analysis of magnetic domains for the pristine and irradiated films has been presented in Figures 1 and 3, respectively. The MFM imaging (Figure 3) along with the integral magnetic measurements (Figure 2) confirmed the modification of magnetic anisotropy, which triggers a spin reorientation transition from OOP to the plane of the film after irradiation. In this section, we are going to discuss the possible changes in domain pattern as a function of *K*_eff_, and *A*_ex_ and qualitative comparison between the simulated and experimental results. It is well known that domain walls (DW) represent the net in-plane component of magnetization, , acting as the boundary between the out-of-plane magnetized (±*M*_*z*_) domains. The width of the DW is proportional to , and hence, the SRT phenomenon can be well understood in terms of the parameters controlling the width of the DW. The simulated domain images are presented in Figure 6a,b for different *A*_ex_ values of 2.3, and 1.15 μerg/cm, respectively, for four different *K*_eff_ values, as mentioned in the left column of the images. For all the domain images of Figure 6, the white and black colors indicate two mutually opposite OOP components of magnetization, and the other colors indicate the IP components, the orientations for which are shown by the arrows. The domain image in Figure 6a with *K*_eff_ = 10 Merg/cm^3^ (top) displays extended maze-like domain structure with strong perpendicular anisotropy. With the reduction in *K*_eff_ to 8 Merg/cm^3^, the domain size decreases, and the pattern appears as extended periodic stripes with no preferential orientations and higher in-plane contrast. With further reduction in *K*_eff_, the extended stripe patterns shrink to circular stripes (not shown) to minimize the magnetostatic energy. Interestingly, strong in-plane contrast leading to asymmetric vortex structure with an out-of-plane magnetized core can be observed with *K*_eff_ = 5 Merg/cm^3^. Further reduction in *K*_eff_ results in a feather-like structure with multiple vortices (bottom). The domain features display significant changes with the reduction of *A*_ex_ to 50% for the same *K*_eff_. Unlike extended maze-like patterns, worm-like domains without preferential orientation can be observed with *A*_ex_ = 1.15 μerg/cm, as shown in Figure 6b. Stronger OOP contrast with reduced domain sizes can be observed with respect to the corresponding domain images of Figure 6a with the same *K*_eff_. Here, the threshold for transition from OOP to IP magnetization can be marked at *K*_eff_ = 5 Merg/cm^3^, where the OOP components shrink to a thread-like region bounded by IP magnetization in a different direction. Further reduction of *K*_eff_ leads to a symmetric vortex configuration. Thus, with the combination of variables *K*_eff_ and *A*_ex_, we can explain tunability in the magnetic domain structure, which is qualitatively similar to the experimentally observed domain images in terms of the extended maze-like pattern in the pristine condition, fragmented thinner domains with reduced OOP magnetization after irradiation at lower fluences, and finally, feather-like structure with dominant IP magnetization in response to the irradiation at higher ion fluences is formed.

In view of the experimental and simulated results, it is evident that a proper understanding of magnetic domain patterns provides a direct route to understanding the change in micromagnetic energetics in response to the physical processes. Thus, it urges to establish a reliable path for proper estimation of important parameters controlling the energetics, which we visualize in the form of magnetic domains. It is important to mention that recognizing the modifications in the micromagnetic parameters from the domain images become extremely difficult with the human eye when the change in parameters does not lead to a significant change in the images, or domain patterns obtained by simultaneous changes in two or more parameters. In this regard, we propose different CNN architectures to identify the micromagnetic parameters from the domain images, which also help immensely to complement the experimental results. As mentioned earlier, training the CNN requires a large and homogeneous data set, which is not easy to obtain through modifications of experimental process conditions. Therefore, in this article, we are using only 960 domain images obtained through micromagnetic simulations considering different combinations of *K*_eff_, *A*_ex_, *T*, and α. The tuning of the domain size and net magnetization can arise from the modification of the abovementioned parameters, but pinpointing the exact parameters with higher accuracy from the entire parameter space is not straight forward. In order to reduce the expense of simulation time and increase accuracy, we have reduced the simulation area to around 1 μm × 1 μm. However, the CNN model is not limited to the size of the images, which we have confirmed by verifying our model with different sizes of images.

### Overview of the CNN

In order to predict the micromagnetic properties (parameters) directly from the domain images, we have used advanced numerical deep learning methods, such as CNN. As we have mentioned in the previous section, CNN consists of a convolutional part associated with a fully or partially connected NN.^69^ Here, we will briefly discuss the fundamental working principle of CNN.

**Figure 7:**
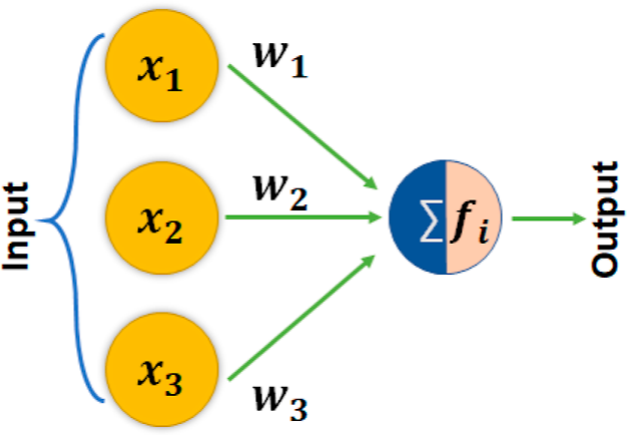
Schematic of an artificial NN with three input and one output neurons. Here, *w*_*i*_, *x*, and *f*_*i*_ are the weights, input values, and threshold function, respectively.

First, we are going to explain different components of the NN and the generally used terminologies. NN primarily consists of node layers that include the input and output layers and hidden layers, which are the dense layers between the input and output layers. Each neuron is connected with other neurons through weights in between and the threshold values. If the output value of a neuron reaches the threshold values, that particular neuron is activated and sends information to the next layer. In general, the weights between different connections can be adjusted by various methods following the back-propagation or forward-propagation algorithms to minimize the error in the output node layer. The simplest model is to consider a NN of 3 input layers and 1 output layer, as shown in the schematic of Figure 7. In this case, we can write the equations for the input layer and the simple threshold or activation function (*f*_*i*_(*x*)) as
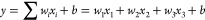
1
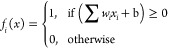
2where *w*_*i*_, *x*_*i*_, and *b* are the weights, input values, and bias, respectively. A bias vector is an additional set of weights in a NN with no input, and thus, it corresponds to the output of an artificial NN with zero input. Once the weights *w*_*i*_ are determined, we can also investigate the contribution of different input properties. In general, larger weights for the input values with comparable magnitude represent a higher contribution to the prediction. Then, the summed function will pass through the activation function, which determines the output. If the output values are higher than the threshold values, the neuron will be activated. This leads to the propagation of the data to the next layer. In this way, finally, we calculate and minimize the cost function or error function, , by adjusting the weights.

**Figure 8:**
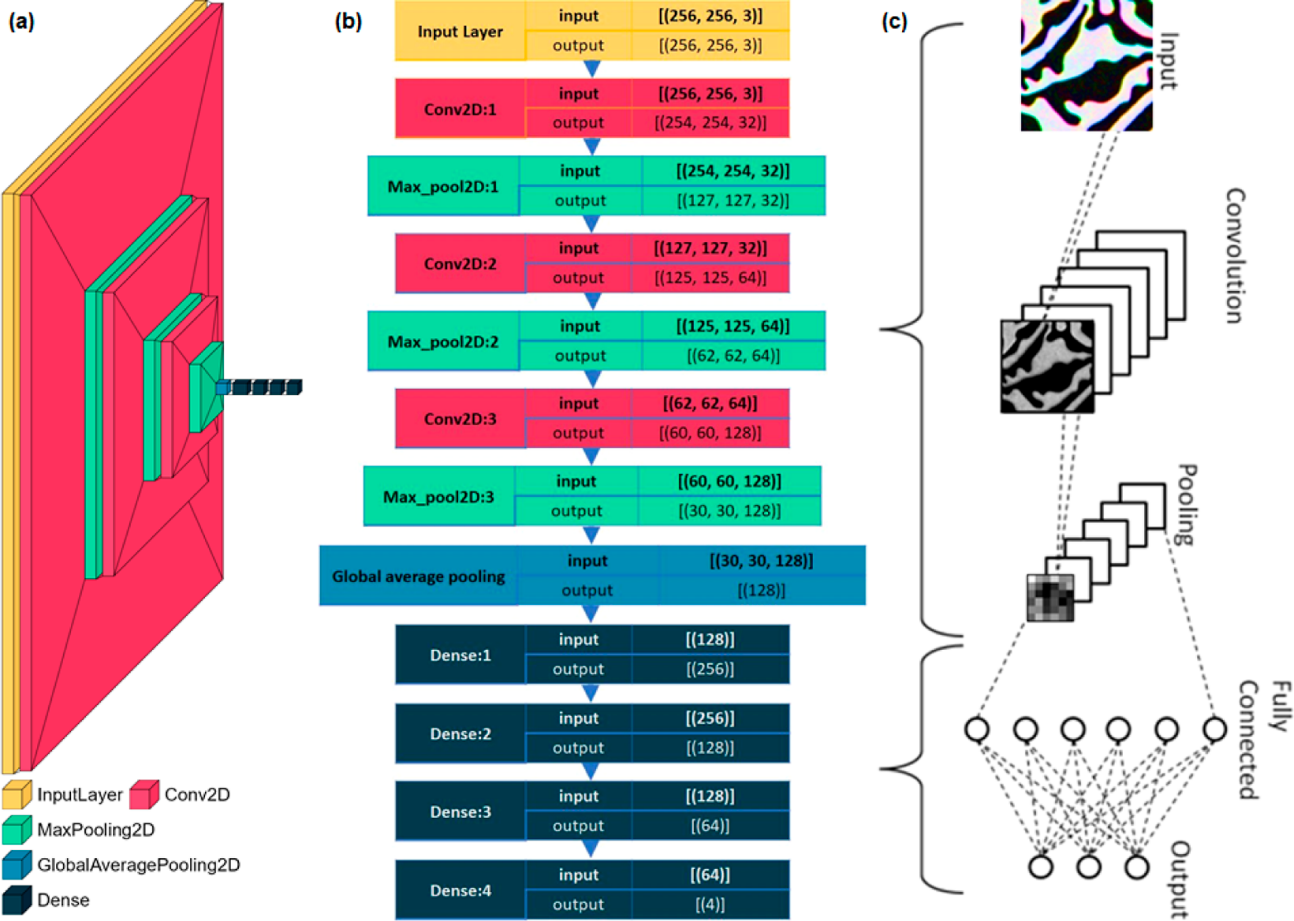
Details of the custom NN with different descriptions: (a) schematic for the reduction in dimension of the input image in each layer of CNN, (b) flow chart of a custom CNN model, and (c) qualitative working principal of the CNN.

**Figure 9:**
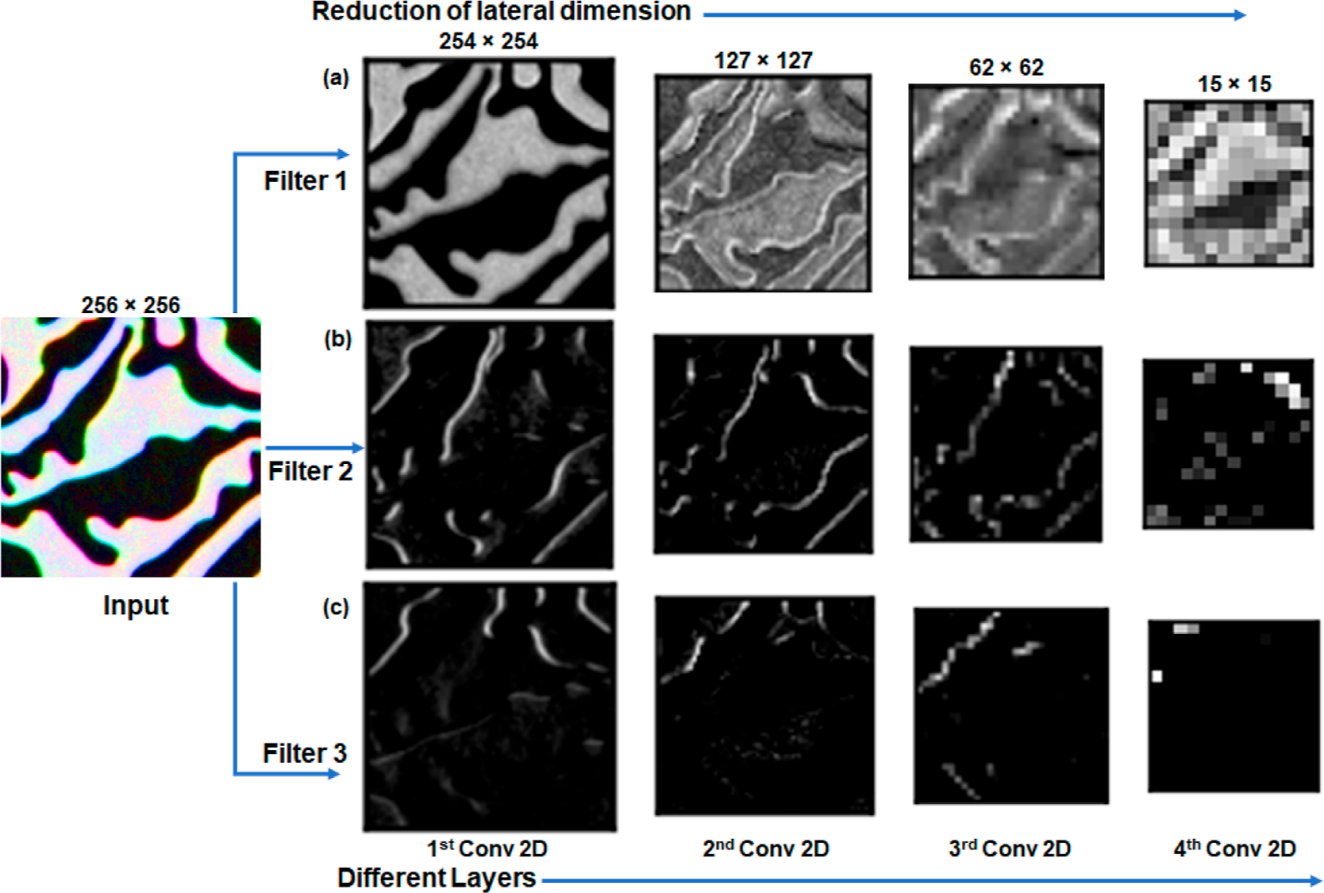
Evolution of images with the application of different filters on the input image and feature extraction from different layers, (a) distribution of out-of-plane magnetization, (b) domain boundaries, and (c) curvature or branching.

Now, while investigating with images, we do not have the exact numerical values of the parameters, mentioned above. Therefore, convolutional layers are used to extract those features from the images.^70^ Thus, in addition to the dense layers, the CNN model contains convolutional layers and pooling layers, which are utilized to extract important features from the images. The schematic for the distribution of different layers in a typical custom CNN is shown in Figure 8a, where three convolution layers and four dense layers were used. In order to extract the features, the convolution layer transforms the input image using the different convoluted filters. A filter is a small matrix with a dimension smaller than that of the image to be convoluted. A single convolution layer contains a series of filters as shown in Figure 8c. Following the convolution layer (Conv2D), we have also used the pooling layer (MaxPooling2D) to reduce the dimensions of the feature maps (Figure 8a), which is helpful in reducing the number of parameters to learn and the amount of computation performed in the network. A reduction in the lateral dimension of the input image can be observed after getting filtered through each layer. While the convolution layer increases the depth effect with a little reduction in the lateral dimension, the pooling layer only contributes to the reduction in lateral dimension. After getting filtered through a series of convolution and pooling layers, the images pass through the global average pooling layer and various dense layers. The sequence of different filters in our custom model and the corresponding changes in dimension at each layer has been well illustrated in the flow chart of Figure 8b. The machine essentially understands and compares the “higher dimensional images” obtained after filtering through all the layers of the CNN model. The features, extracted using the filters, were further used for the prediction or classification. The entire process of working principal of CNN can be understood through the schematic of Figure 8c. However, in practice, substantially more convolutional and pooling layers are used in a CNN to extract different features from the images. In order to understand the effect of filters inside different convolution and pooling layers, we have shown an example of image regression in Figure 9, illustrating the action of filtering three important features when the simulated domain image of Figure 6a pass through different filters in different convolution and pooling layers. In Figure 9a, the first filter (top row) is extracting the distribution of the out-of-plane magnetization, whereas the second filter (middle row) in Figure 9b marks the domain walls, which are the boundaries between the two oppositely magnetized areas, and the third filter (bottom) in Figure 9c brings information about the domain curvature, which is physically related to domain nucleation and branching. However, the information extracted in the last layer (4th Conv2D layer and higher) from a specific filter in Figure 9 becomes too complex to understand with the human eye. Several other filters were also used to produce this higher dimensional information, which was further fed to the NN to identify particular features inside the image, where we use different functionalities mentioned above to predict the magnetic properties. It is important to notice that the lateral dimension of the images decreases from 256 × 256 to 15 × 15 after passing through all the filters, as observed from Figure 9.

**Figure 10:**
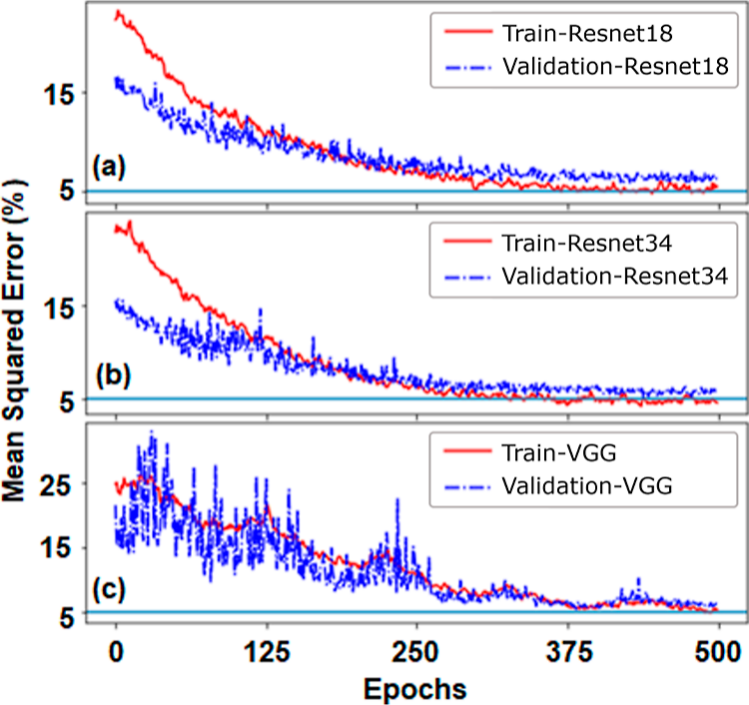
Evolution of the loss function for different CNN models as a function of epochs. Here losses for training and validation images are shown by solid and dashed lines, respectively. The blue solid line represents a reference level at 5%.

Now, we will look into the training process of the CNN model. For the training, we use particular images, grouped with batch sizes of 32 images. Batch size decides the number of images trained at a time. We trained the CNN model on the entire training data set, also known as epochs. After training the model, we checked the prediction of the model to validate images and calculated the mean squared error (MSE) for each epoch. Based on the errors, the model tries to adjust the weights to reduce the MSE. We have used different models for training, and their comparative performances are shown in Figure 10, where the MSE has been plotted at different epochs. A large MSE difference between the trained and validation data sets indicates overfitting in the model. As observed from Figure 10, the difference between the validation data set and the trained data set is small, which implies that the models are learning different features from the images. Further, the three different models are converging with comparable values of MSE (close to 5%) for higher epochs. We have tried to estimate the magnetic properties from an experimental domain image, which has been shown in Figure S4 of the Supporting Information.

#### Comparison with Pretrained CNN Architectures

We have constructed our custom CNN architecture and compared it with different available CNN architectures. Many numerical methods, such as Pearson correlation coefficient, mean squared error (MSE), *R*^2^ score, and so forth, are used to check the accuracy of the prediction. In our case, we have checked the accuracy of each model by the goodness parameter, *R*^2^ score, as defined in the eq 3. It is a measure of fit that indicates the variation of a dependent variable, explained by the independent variable(s) in a regression model. Furthermore, we also report different *R*^2^ scores for different methods in Table 2. Here, it is worth mentioning that the reported *R*^2^ scores are calculated using the test images, which were never exposed to the model during the training process.
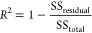
3

**Table 2:** Comparison of the *R*^2^ Scores and Time of Training for 1000 Epochs (500 Epochs with Each of Freeze and Unfreeze Weights) for Different CNN Models^a^

name of the model	*R*^2^ score (%)	training time (s × 10^3^)
custom model [Figure 8]	81.8	5
ResNet18	90.2	130
ResNet34	93.4	145
VGG16	93.9	72
EfficientNet	92.9	65
AlexNet	89.1	35
DenseNet	91.1	75

a The time for training is based on an Intel i5 quad core processor.

In eq 3,  and , where *f*_*i*_ are the predicted values; *x*_*i*_ and *y*_*i*_ are values of input and output properties, respectively; and *x̅* and *y̅* are the mean of the input and output values, respectively. In general, the values of *R*^2^ score should lie between 0 and 1, where 0 indicates no dependency of input parameters on output properties (least accuracy), while 1 shows the 100% dependence (maximum accuracy). Negative values of *R*^2^ score are also possible, indicating the worst performance by selecting the mean value from the data set.

In addition to that, we refer to Table 2, which shows that the pretrained models that have a significantly large number of layers than the custom model work comparatively better. We run each pretrained model in two parts: (1) using the freeze (unchanged) weights and (2) using the unfreeze weights, as suggested in the model manual.^53^ It is a general practice to decrease the load on the computer and preserve the prefixed weights in pretrained models since these CNN architectures have around half a million parameters to adjust. We can see significant improvement when we increase the layers from the custom model to ResNet18, after which the improvement is not significant while increasing the number of layers in ResNet34. A larger number of layers generally captures higher-dimensional features from the images; however, after some threshold number of layers in the model, the possibility of overfitting increases.

## Conclusions

This paper explains a detailed understanding of modifications of magnetic domains in the perpendicularly magnetized Co/Pd ML. Sputtered ML films with *t*_Co_ = 3 Å show the highest PMA with maze-like magnetic domains in pristine condition. The integral magnetic properties and magnetic domains are influenced significantly by the irradiation of Ar^+^ ions at 50 keV of energy. The magnetic contrast decreases, and the domain size reduces to almost one-third of that in the pristine state after the bombardment of ions with a fluence of 10^14^ ions/cm^2^. Irradiation with a higher ion fluence renders the effective anisotropy to reorient along the plane of the film. As a result of that, no definite periodic domain configurations were observed. Instead, feather-like domain patterns with localized vortices could be seen. The changes in magnetic properties can be corroborated by the structural modifications in terms of diffusion and alloying at the interfaces of the ultrathin Co and Pd layers. In this context, we felt an urge to establish a reliable and automated way to predict the magnetic properties directly from the microscopic investigation. Advanced machine learning algorithms, in the form of a CNN, were found to be the best route to achieve our goal. In order to produce a large data set of images to train the CNN, micromagnetic simulation was used, which acts as a bridge between microscopy and the CNN, as the simulated domain images showed good qualitative agreement with the MFM images. The CNN architecture, trained through 960 simulated images, successfully predicts the micromagnetic parameters with a maximum efficiency of 93.9%, which is quite high and well comparable with the recently reported results. However, the model requires the same lateral dimension of images and similar color information while comparing the trained and validation data sets. From the point of view of a realistic change in magnetic properties correlated with the experimental results, we have considered here the variations of *K*_eff_, *A*_ex_, *T*, and α to create the data set for training. In the future, the CNN model can be extended to a more complex form, extending to the variations of *M*_s_, external magnetic field, DMI constant, and spin-polarized current, encompassing the aspect of predicting novel magnetic materials with chiral domain wall/skyrmion configurations, to be manipulated using the current..

## Supporting Information Available

Hysteresis loops, AFM and MFM images, and XRR spectra of the samples irradiated at an energy of 100 keV of the Ar^+^ ions and estimation of magnetic properties from the MFM image (PDF)
