# Supplementary material for: Understanding the Magnetic Microstructure through Experiments and Machine Learning Algorithms
Source: ACS Appl Mater Interfaces. 2022 Oct 21;14(44):50318–30. doi: 10.1021/acsami.2c12848 (PMC9650662; doi:10.1021/acsami.2c12848)
Supplement: Supplementary file 1 — am2c12848_si_001.pdf [file am2c12848_si_001.pdf]

Supporting Information
Understanding Magnetic Microstructure through Experiments and Machine Learning Algorithms

Abhishek Talapatra,[*,†,§] Udaykumar Gajera,[*,‡,‖] Syam Prasad P,[†] Jeyaramane Arout Chelvane,[¶] and Jyoti Ranjan Mohanty[*,†]

†*Nanomagnetism and Microscopy Laboratory, Department of Physics, Indian Institute of Technology Hyderabad, Kandi, Sangareddy 502285, Telangana, India*

‡*Consiglio Nazionale delle Ricerche, CNR-SPIN c/o Università "G. D'Annunzio", 66100 Chieti, Italy*

¶*Defence Metallurgical Research Laboratory, Kanchanbagh, Hyderabad 500058, India*

§*Present Address: Department of Applied Physics, Aalto University School of Science, FI-00076 Aalto, Finland*

‖*Chemistry Department, University of Turin, via Pietro Giuria, 7, 10125, Torino, Italy*

E-mail: atalapatra89@gmail.com; uday.gajera@edu.unito.it; jmohanty@phy.iith.ac.in

Magnetic and Structural Characterizations of the Films Irradiated with Ar^+ Ions of Energy 100 keV

We are going to complement here the results provided in the main manuscript for the films irradiated with the Ar^+ ions of energy (E) 50 keV. Here, we are presenting the data for the irradiated films at $E = 100\ keV$ for different fluences.

Hysteresis Loops

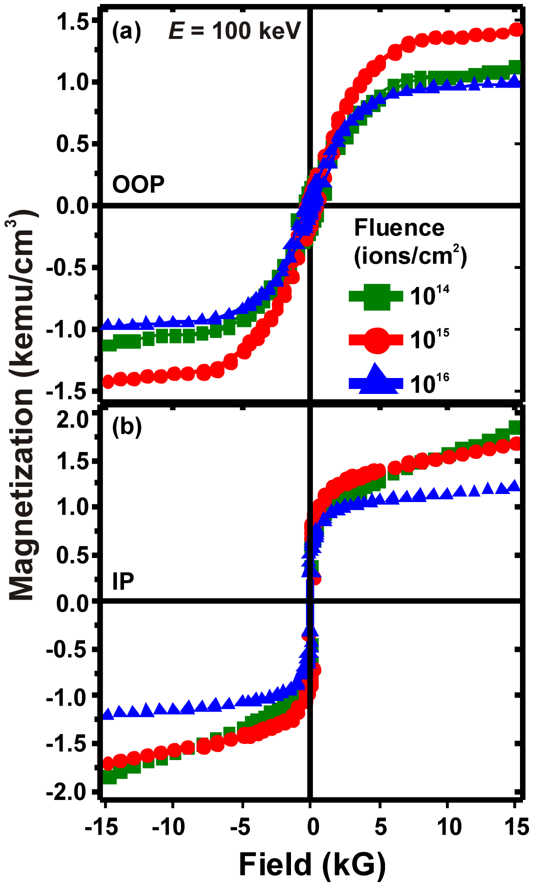

Figure S 1: Hysteresis loops of the irradiated films with applied field along the (a) out-of-plane and (b) in-plane directions.

The variations in the out-of-plane (OOP), and in-plane (IP) hysteresis loops for the films irradiated with Ar^+ ions of energy 100 keV are shown in Fig. S1 (a), and (b) respectively. The hysteresis loops are qualitatively comparable to that for the films irradiated with $E = 50$

kev (Fig. 2 of the main manuscript). Due to the larger penetration depth of the bombarded ions at higher E, comparatively smaller fluence is sufficient to tune the effective anisotropy K_{eff}. Slanted OOP loops with negligible remanence and higher saturation field (Fig. S1 (a)) in comparison with the almost rectangular IP loops (Fig. S1 (b)) clearly signify the reduction in perpendicular magnetic anisotropy (PMA) at an increased ion energy.

Atomic and Magnetic Force Microscopy

The atomic and magnetic force microscopy (AFM and MFM) images are depicted in Fig. S2 (a) and (b) respectively. The estimated root mean squared roughness (R_q) appears to be larger compared to that observed from Fig. 3 (a) of the main manuscript. The maximum R_q was 6.5 *nm* at the maximum fluence. The fragmented magnetic domains, observed at lower fluences (Fig. 3 (b) of the main manuscript) are no longer observed for the films irradiated with higher energy. Instead, in-plane variation of magnetic contrast in the form of feather-like domains with cross-type walls can be clearly observed for first four MFM images (from left) of Fig. S2 (b). Due to the higher roughness at maximum fluence, the topography-induced mixed phase can be seen in the MFM image.

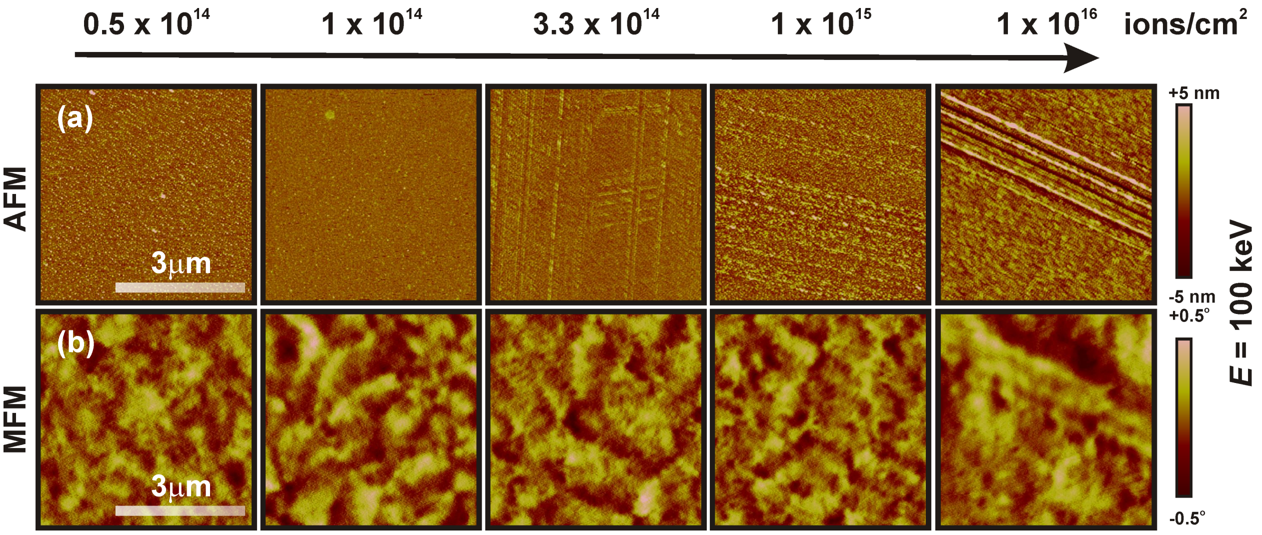

Figure S 2: Series of normalized (a) AFM images with simultaneously captured corresponding (b) MFM images for the films irradiated with 100 *kev* of Ar^+ ions at various fluences (mentioned on top of the image). The scale bar is same for all the images.

X-ray Reflectivity

The experimental and fitted X-ray reflectivity (XRR) spectra are shown in Fig. 3 for the films irradiated with $E = 100\ keV$ at two different fluences (F), 0.5×10^{14} (minimum), and $3.3 \times 10^{16}\ ions/cm^2$ (maximum). The best-fitted model reveals that the anticipated pristine structure $[Co(3)/Pd(8)]_{\times 50}$ (the thickness values are in $\AA$) of the multilayer film gets modified to $[Co(1.49)/CoPd(3.61)/Pd(5.18)]_{\times 50}$ with an average interfacial roughness around 0.44 $\AA$ for the films irradiated at 100 keV energy with minimum fluence. With the increase in F, the experimental data shows the decay of the spectral oscillations within a narrow range of 2θ. The fitting suggests a complete diffusion of Co in Pd resulting in a modified structure in the form of $[CoPd(12.49)/Pd(1.8)]_{\times 50}$ with an increased interfacial roughness around 1.56 $\AA$. It is worth mentioning that the fitted XRR model also suggested the presence of a thin $CoPd$ alloy layer due to interfacial diffusion for the pristine film, as shown in Fig. 5 of the main manuscript.

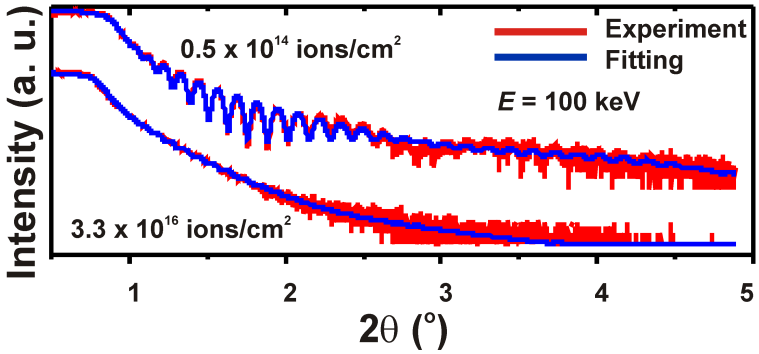

Figure S 3: Experimental and fitted XRR spectra for the films irradiated with 100 keV ion energy at two different fluences.

Estimation of Magnetic Properties from the Experimental Domain Image Using Convolutional Neural Network

As mentioned in the main manuscript, we have generated almost 960 images using micromagnetic simulations by changing the combinations of four input parameters namely, K_{eff},

exchange constant (A_{ex}), Temperature (T), and damping constant (α). Training, testing, and validating with the simulated domain images provided a maximum accuracy of 93.9% (Table 2 of the main manuscript). The color information in the MFM images are different compared to that of the simulated images. Moreover, the Fourier transform filter was applied to the original MFM images to get rid of the tip-induced artefacts. Despite the dissimilarities, we tried to compare the simulated and experimental images at the same footing by converting those into the grayscale format. In order to do that, we trained the convolutional neural network (CNN) separately with the simulated domain images in grayscale format. Now, the MFM image in Fig. 1 (e) of the main manuscript was tested with the CNN which provided the values of the magnetic properties as, $K_{eff} = 23.9\ Merg/cm^3$, $A_{ex} = 19.6\ \mu erg/cm$, $T = 316\ K$, and $\alpha = 0.9$. The same set of values were used for the domain simulations. The results are presented in Fig. S4, displaying the experimental and simulated domain images in grayscale format. The simulated domain image is qualitatively comparable with the experimental domain images in terms of nanoscale, periodic, interconnected maze-like domains with comparable dimensions, the curling and branching of domains highlighting the probability for nucleation dominated reversal, and the presence of in-plane domain walls.

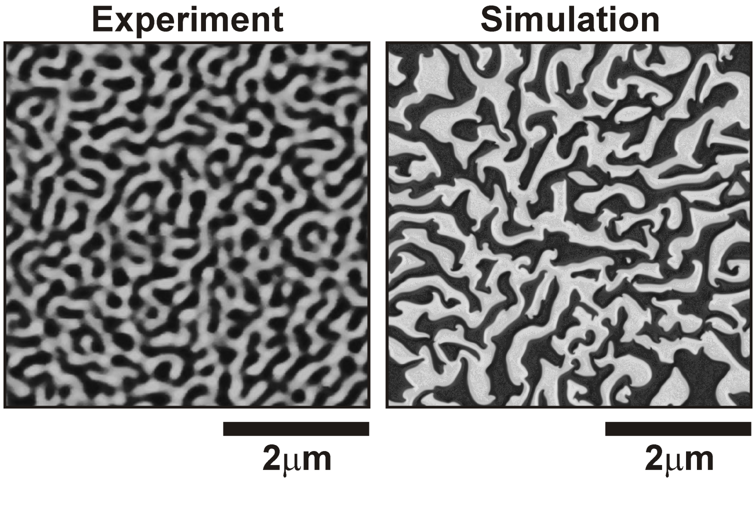

Figure S 4: Experimental and simulated domain images, obtained with the input parameters derived from the image regression algorithm, applied on the experimental domain image.
